# What do we know about the risks for young people moving into, through and out of inpatient mental health care? Findings from an evidence synthesis

**DOI:** 10.1186/s13034-015-0087-y

**Published:** 2015-12-23

**Authors:** Deborah Edwards, Nicola Evans, Elizabeth Gillen, Mirella Longo, Steven Pryjmachuk, Gemma Trainor, Ben Hannigan

**Affiliations:** School of Healthcare Sciences, College of Biomedical and Life Sciences, Cardiff University, Cardiff, UK; Information Services, Cardiff University, Cardiff, UK; College of Human and Health Sciences, Swansea University, Swansea, UK; School of Nursing, Midwifery and Social Work, University of Manchester, Manchester, UK; Greater Manchester West Mental Health NHS Foundation Trust, Manchester, UK

**Keywords:** Contagion, Child and adolescent mental health services, Education, Families, Friends, Identity, Inpatient, Normal life, Risk, Stigma

## Abstract

**Background:**

Young people with complex or severe mental health needs sometimes require care and treatment in inpatient settings. There are risks for young people in this care context, and this study addressed the question: ‘What is known about the identification, assessment and management of risk in young people (aged 11–18) with complex mental health needs entering, using and exiting inpatient child and adolescent mental health services in the UK?’

**Methods:**

In phase 1 a scoping search of two electronic databases (MEDLINE and PsychINFO) was undertaken. Items included were themed and presented to members of a stakeholder advisory group, who were asked to help prioritise the focus for phase 2. In phase 2, 17 electronic databases (EconLit; ASSIA; BNI; Cochrane Library; CINAHL; ERIC; EMBASE; HMIC; MEDLINE; PsycINFO; Scopus; Social Care Online; Social Services Abstracts; Sociological Abstracts; OpenGrey; TRiP; and Web of Science) were searched. Websites were explored and a call for evidence was circulated to locate items related to the risks to young people in mental health hospitals relating to ‘dislocation’ and ‘contagion’. All types of evidence including research, policies and service and practice responses relating to outcomes, views and experiences, costs and cost-effectiveness were considered. Materials identified were narratively synthesised.

**Results:**

In phase 1, 4539 citations were found and 124 items included. Most were concerned with clinical risks. In phase 2, 15,662 citations were found, and 40 addressing the risks of ‘dislocation’ and ‘contagion’ were included supplemented by 20 policy and guidance documents. The quality of studies varied. Materials were synthesised using the categories: Dislocation: Normal Life; Dislocation: Identity; Dislocation: Friends; Dislocation: Stigma; Dislocation: Education; Dislocation: Families; and Contagion. No studies included an economic analysis. Although we found evidence of consideration of risk to young people in these areas we found little evidence to improve practice and services.

**Conclusions:**

The importance to stakeholders of the risks of ‘dislocation’ and ‘contagion’ contrasted with the limited quantity and quality of evidence to inform policy, services and practice. The risks of dislocation and contagion are important, but new research is needed to inform how staff might identify, assess and manage them.

## Background

In Britain it is estimated that one in ten children and young people aged between five and sixteen has a diagnosable mental health problem [1]. Services are organised using a tiered approach, with the most specialist and intensive care (often provided in hospitals) available at tier 4 for children and young people with the highest levels of need. Typically, decisions on who to admit to inpatient child and adolescent mental health services (CAMHS) take place in conditions of scarce resources, with perceptions of ‘risk’ uppermost. Whilst reasons for admission to hospital are complex, inpatient care is often selected because the round-the-clock availability of staff makes it possible to meet needs in comprehensive fashion and to keep young people safe.

Keeping young people safe means that the risks of suicide, self-harm and self-neglect and the risks of harm to others are vital considerations in the CAMHS system context. However, these are not the only risks facing young people experiencing mental health difficulties and their families. The evidence synthesis summarised in this paper was designed with a broad view of ‘risk’ in mind, recognising that risk is both complex and multifaceted. The lived experience of mental ill-health and admission to hospital pose risks to young people’s psychosocial development, their educational achievement, and family and peer relations. In this context the overarching research question in the study reported here was:

What is known about the identification, assessment and management of risk (where ‘risk’ is broadly conceived) in young people (aged 11–18) with complex mental health needs entering, using and exiting inpatient child and adolescent mental health services in the UK?’

This article summarises methods and key findings, derived from a full report of the study [2]. The article particularly focuses on methods and findings in the in-depth second phase of the larger study.

## Methods

**Fig. 1:**
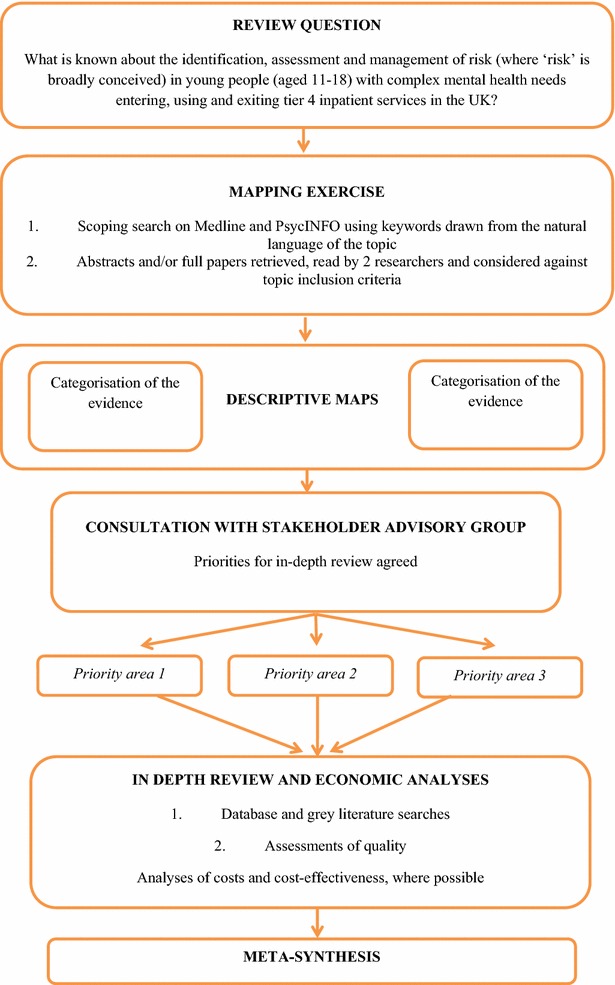
The EPPI-Centre approach used in this study

The two-stage Evidence for Policy and Practice Information and Co-ordinating Centre (EPPI-Centre) approach was used [3]. Figure 1 summarises how the EPPI-Centre approach was used in this study.

### Phase 1 scoping: methods and findings as a precursor to in-depth phase 2

**Fig. 2:**
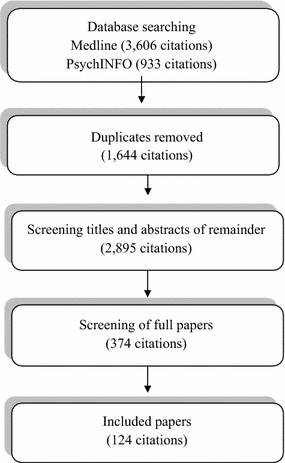
Flow of studies in phase 1

In the first phase, two databases (MEDLINE and PsychINFO) were searched using clear criteria for the inclusion of citations: English language; focusing on young people aged 11–18 making the transition through inpatient mental health services; and concerned with risk identification and/or risk assessment and/or risk management (where ‘risk’ was not defined in advance by the project team). In addition, as not all citations retrieved were clear in describing types of service, ‘inpatient mental health services’ was defined as any inpatient hospital services (and, in the case of US citations, residential treatment centres) staffed by mental health professionals. Of 4539 citations retrieved (none of which were subjected to quality appraisal) 124 citations were included (see Fig. 2 for flow of studies).

**Fig. 3:**
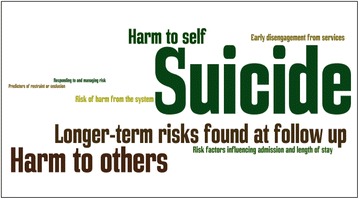
Phase 1 themes

These were summarized in a series of maps focusing on ‘harm to self’, ‘suicide’, ‘harm to others’, ‘longer-term risks found at follow-up’, ‘early disengagement from services’, ‘risk factors influencing admission and length of stay’, ‘predictors of restraint or seclusion’, ‘risk of harm from the system’, ‘responding to and managing risk’ and ‘other’. The themes identified are presented in Fig. 3, where the size of each word reflects the number of articles grouped in each category.

In parallel to the electronic search, a collaborator working for the national charity YoungMinds conducted consultative conversations with five young people previously admitted to inpatient CAMHS. Conversations were recorded, and young people were asked to identify risks which the project team should focus on in phase 2 of the project. A summary of these conversations was written up. A similar consultative conversation took place with the mother of a child who had been admitted to inpatient CAMHS.

Descriptive maps of the findings from phase 1 were presented to a stakeholder group which included CAMHS managers, practitioners from different backgrounds, young people, a key collaborator from YoungMinds [n = 7] and all members of the project team [n = 7]. Informed by the principles of nominal group technique [4], participants generated independent lists of the risks for young people making the transition into, through and out of inpatient mental health care. These were collated and displayed. Participants then ranked, in writing, their personal priorities for the categories of risk to take forward into the second, in-depth, phase of the project.

Stakeholders’ priority categories of risk were combined with the priorities previously identified from the YoungMinds consultations. Items were coded and themed, and a list of ranked priority risk categories created. This list was circulated to the stakeholder group for a final round of comments.

### Priority areas identified

Priorities were grouped under the umbrella terms ‘dislocation’ and ‘contagion’. These terms were created by the project team, based on an inductive reading of the items contained in the priorities list produced in the context of the consultative conversations and stakeholder consultation, and without specific regard to the themes identified in phase 1 (and reproduced in Fig. 3). ‘Dislocation’ was the term used by the project team to refer to the risks: of being removed from normal life; to identity; of stigma; to friendships; to families; to education; to psychological development; and to social development. ‘Contagion’ was used to refer to the risks of learning unhelpful behaviour and making unhelpful friendships.

### Phase 2 methods: in-depth review of prioritised risks

Phase 2 centred on the search, appraisal and synthesis of English-language citations relating to the risks to young people in these prioritised areas. The final search strategy used was highly sensitive and comprised three core concepts: (1) young people; (2) mental health; and (3) inpatient. Searches were made using the following 17 databases, with time limits from 1995 to September 2013: EconLit (American Economic Association’s electronic bibliography); Applied Social Sciences Index and Abstracts; British Nursing Index; Cochrane Library; Cumulative Index to Nursing and Allied Health Literature; Education Resources Information Center; EMBASE; Health Management Information Consortium; MEDLINE; PsycINFO; Scopus; Social Care Online; Social Services Abstracts; Sociological Abstracts; OpenGrey; Turning Research into Practice Plus; and Web of Science. The project team reviewed all citations retrieved and manually identified those addressing the risks of dislocation and contagion, and extracted data using an abstraction document designed for the study. Care was also taken at this stage to include any citations addressing costs and cost-effectiveness. UK government and other organisational websites were searched, in order to include contextual information (e.g., policy drivers) and as a route to the identification of additional evidence. A call for evidence was circulated, and references of included citations were reviewed.

All types of evidence relating to outcomes, views and experiences, costs and cost-effectiveness, policies, and service and practice responses in the areas of ‘dislocation’ and ‘contagion’ for young people using inpatient mental health services were considered. A staged approach to screening and selection of citations was used, involving all members of the project team. Data from all included citations were extracted into tables formatted following guidance issued by the Centre for Reviews and Dissemination [5] or into tables developed for the purpose of the review. Quality of research items included in phase 2 was appraised using relevant checklists [6–9].

Given the heterogeneity of the items included in phase 2, all materials were brought together in a series of individual narrative syntheses [10] each reflecting an a priori area of risk previously identified. Sub-categories were created inductively [11]. The strength of synthesised findings for phase 2 (intervention studies) was assessed using the Grading of Recommendations Assessment, Development, and Evaluation (GRADE) approach [12] where certainty of evidence is reported as being high, moderate or low/very low. Confidence in synthesised qualitative and survey findings was assessed using the Confidence in the Evidence from Reviews of Qualitative research (CERQual) tool, which uses a similar approach to GRADE [13]. The original CerQual approach was designed for qualitative findings and we used the same process but included findings from surveys in the assessment of confidence. Confidence in findings is described as high, moderate or low. No quality assessment was undertaken for policy and guidance documents. Similarly, no methodological quality assessments were conducted for the reports of local service or practice developments, or the case reports.

## Results

**Fig. 4:**
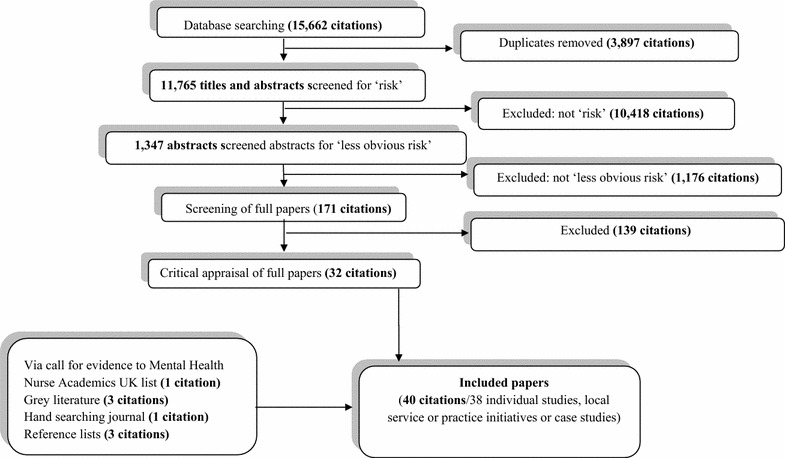
Flow of studies in phase 2

In phase 2 a total of 15,662 citations were identified in the database searches (see Fig. 4 for search results and study selection). Forty papers (reporting on 38 studies) were included in the final review, along with a total of 20 policy and guidance documents specifically addressing the CAMHS field, or assessed as otherwise including material directly relevant to the aims of the study.

### Description of the included studies

Information on the characteristics of included studies, including assessments of quality, is given in Table 1.

**Table 1:** Included studies in phase 2

Author (s) year, Country	Participants	Setting	Length of stay (days)	Age (years)	Gender (% male)	Focus	Quality
Prospective cohort study							
Best et al. [14], USA	Hospital: Ad (n = 70) High school: Ad (n = 76)	Psychiatric units (n = NS) matched with one high school in same area	Range 37–921; mean 198	Hospitalised: mean 14.1 ± 1.0; range 11.8–15.9 High school: mean 14.5 ± 0.4; range 13.8–15.8	Hospitalised: 56 High school: 46	Dislocation: Ed	Strong
Before-and-after study (no control group)							
Singh et al. [16], USA	Study 1, HCP: pre (n = 18); post (n = 18) Study 2, HCP (n = 5)	Study 1: inpatient unit (n = 1) Study 2: inpatient unit (n = 1)	Study 1: NA Study 2: NA	Study 1: NA Study 2: NA	Study 1: NA Study 2: NA	Study 1: Dislocation: Fa Study 2: Dislocation: Fa	Study 1: weak Study 2: weak
Quasi-experimental multiple time series (retrospective)							
Simmerman [15], USA	Ad (n = 107)	RTC (n = 1)	NS	Range 12–19 years	NS	Dislocation: Ed	Weak
Qualitative studies							
Buston [27], UK (Scotland)	Ad (n = 12)	Previously inpatients	NA	Mean 17.3; range 14–20	34	Dislocation: Fa, Fr, Ed	A, B, C, E, F, G, H, I High
Claveirole [18], UK (Scotland)	Ad (n = 18); P (n = 16); HCP (n = 23)	Inpatients (n = NS)	NS	Mean 16; range 12–21	33	Contagion Dislocation: Fa, Fr, NL, St, Ed	A, B, C, D, E, F, G, H, I High
Clemens et al. [28, 48], USA	HCP (n = 14)	Inpatient units (n = 4)	Range 5–7	NA	NA	Dislocation: Ed, Fr	A, B, C, E, F, G, H, I High
Colton and Pistrang [26], UK (England)	Ad (n = 19)	Inpatient units (n = 2), eating disorders	Range 35–140	Mean 15.4; range 2–17	0	Contagion Dislocation: Fa, Id, Fr	A, B, C, D, E, F, G, I, High
Haynes et al. (2011)/Painter [19], UK (England)	Ad (n = 10)	Inpatient units (n = 2)	Range 4 weeks to 36 months	Range 13–19	40	Dislocation: Fr, St, Ed, NL, Id	A, B, C, E, F, G, H, I High
Moses [20], USA	Ad (n = 80)	Inpatient unit (n = 1)	Mean 7.6 ± 3.9; range 3–31	Mean 15.3; range 13–18	59	Dislocation: Fr, NL, St	A, B, C, D, E, F, G, H, I High
Offord et al. [21], UK (England)	Ad (n = 6)	Inpatient units (n = 6), eating disorders	NS	Range 16–23	0	Contagion Dislocation: Fa, Id, Fr, Ed, St, NL	A, B, C, D, E, F, G, H, I High
Puotiniemi and Kyngas [29], Finland	Ad (n = 1); P (n = 1)	Previously an inpatient	NS	16	0	Dislocation: Fa, Fr	A, B, C, E, F, H Medium
Longitudinal descriptive survey (prospective)							
Anderson et al. [49], USA	Ad (n = 424); FU (n = NS)	Inpatient units (n = 5), substance abuse	NS	Mean 15.9 ± 1.3; range 13–18	49.5	Dislocation: Ed	A, B, C, D, E, H Medium
Brinkmeyer et al. [50], USA	P (n = 47); FU-1 (n = 34); FU-2 (n = 25)	Inpatient unit (n = 1)	Median 6	Mean 13.11 ± 2.89; range 7–17	56	Dislocation: Fa, Ed	A, B, C, D, E, F, I Medium
Charlemagne [60], USA	P (n = 167); FU (n = 134); HCP (n = 27)	Inpatient unit (n = 1)	Mean 5.9 ± 3.4	Range 13 ± 2.7	48.5	Dislocation: Fa	A, B, C, D, E, F, G, H, I High
Czyz et al. [30], USA	Ad (n = 448); FU (n = 338)	Inpatients (n = NS)	NS	Mean 15.6 ± 1.41; range 13–17	29	Dislocation: Fa, Fr	A, B, C, E, F, G, H, I High
Larzelere et al. [51], USA	Ad (n = 43); FU (n = 43)	RTC (n = 1)	Range 18–505; mean 181; median 15	Mean 13.0; range 6–17	48	Dislocation: Ed	B, C, E, F, I Low
Moses [20], USA	Ad (n = 102); FU (n = 80)	Inpatient unit (n = 1)	NS	Mean 15.3 ± 1.6; range 13–18	35	Dislocation: St	A, B, C, D, E, F, G, H, I High
Parmelee et al. [61], USA	Ch and Ad (n = 90); FU (n = 77)	Inpatient unit (n = 2)	NS	Mean 2.2 ± 3.5; range 4–17	64	Dislocation: Fa	A, B, C, D Low
King et al. [30], USA	Ad (n = 57); FU (n = 57)	Inpatient unit (n = 1)	Mean 24.9 ± 10.7	Mean 15.8 ± 1.3; range 11.5–17.8	42	Contagion	A, B, C, E, F, G, I Medium
Longitudinal descriptive surveys (retrospective)							
Cawthorpe et al. [69], Canada	Ad (n = 198)	Inpatient unit (n = 1)	Males: mean 95.67 ± 56.87; range 1–337 Females: mean 79.82; range 3–254	Males: mean 14.89 ± 1.28; range 12–18 Females: mean 14.77 ± 1.17; range 12–18	64	Contagion	A, B, C, D, E, F, I Medium
Shabat [52], USA	Ch and Ad (n = 457)	RTC (n = 50)	Mean 90.6	Range 6–20	61	Dislocation: Ed	A, B, C, E, F, G, H, I High
Lakin et al. [62], USA	Ch and Ad (n = 89)	RTC (n = 1)	Mean 58.6 ± 35.1; range 7–243	Mean 11.7; range 5–17	67	Dislocation: Fa	A, B, C, D, E, F, H, I High
Halfon et al. [47], France	Ad (n = 137)	Inpatient unit (n = 1)	Mean 10 months 10 days ± 9.3	Mean 17.8 ± 1.5; range 14–21	54	Dislocation: Ed	A, B, C, D, F, I Medium
Cross-sectional descriptive surveys							
Moses [20], USA	Ad (n = 102)	Inpatient unit (n = 1)	Mean 7.6 ± 4.2; range 3–31	Mean 15.3 ± 1.5; range 13–19	91.3	Dislocation: St	A, B, C, D, E, F, H, I High
Thurber et al. [63], USA	Ch and Ad (n = 50); P (n = 75); HCP (n = NS)	Burn unit (n = 1); paediatric rehabilitation unit (n = 1); inpatient psychiatric unit (n = 1)	Median 20; mean 24 ± 25; range 4–174	Mean 11 ± 3.5; range 3.9–18.6	70	Dislocation: Fa	A, B, C, E, G, I Medium
Longitudinal descriptive survey (prospective)							
Taiminen et al. [70], Finland	Ad (n = 12)	Inpatient unit (n = 1)	Mean 91.9 ± 73.2; range 7–200	Mean 15.5; range 12.3–17.9	0	Contagion	A, B, C, D, E, I Medium
Grey literature reports							
Street and Svanberg (2003), Svanberg and Street [22], UK (England and Wales)	Ad (n = 107); P (n = 35); HCP (n = 115); interviews; questionnaire	Inpatient units (n = 10); paediatric wards (n = 2); adult psychiatry ward (n = 1); ‘one-stop ‘service for young homeless people (n = 1)	NS	Mean 16.4	32	Contagion Dislocation: Fa, Fr, NL, Ed	A, B, C, D, E, F, H, I High
Tulloch et al. [46], UK (England)	HCP (n = 42); questionnaire	Independent and public CAMHS in England (n = 42 of 55, 76 %)	Median 79	Median: 12 years (lower end of the range – 38 % of units); 18 years (upper end of the range – 77 % of units)	NS	Dislocation: Ed	A, B, C, D, E, F, G, H, I High
Tulloch et al. [46], UK (England)	Ad (n = 19); P (n = 12); interviews; questionnaire	Independent and public CAMHS in England (n = 42 of 55, 76 %)	NA	NA	NA	Dislocation: Fa	A, B, C, D, E, F, G, H, I High
O’Herlihy et al. [45], UK (England and Wales)	CAMHS units (n = 66); questionnaire	Independent and public CAMHS in England and Wales (n = 66 of 80, 82.5 %)	NS	Mean 115 ± 181; range 0–2194	NS	Dislocation: Ed, Fa	A, B, C, D, E, F, G, H, I High
O’Herlihy et al. [45], UK (England and Wales)	HCP (n = 245); questionnaire	Independent and public CAMHS in England and Wales (n = 80)	NA	NA	NA	Dislocation: Ed A	A, B, C, D, E, F, G, H, I High
Mental Welfare Commission for Scotland [17], UK (Scotland)	Ad (n = 29); HCP (n = 11); interviews; case note review	All independent and public CAMHS in Scotland (n = NS)	NS	NS	NS	Dislocation: Ed, Fa	The study method is not fully detailed (e.g. sampling recruitment, participants age, gender, type of analysis)

*Ad* adolescent, *Adm* admission, *CAMHS* children and adolescent mental health services, *Ch* child, *Ed* education, *Fa* family, *Fr* friends, *FU* follow-up, *HCP* health-care professional, *Id* identity, *NA* not available, *NL* normal life, *NS* not stated, *P* parent, *St* stigma

Quality key: A, clear statement of the aims of the study; B, adequate description of the context for the study; C, clear specification of research design and its appropriateness for the research aims, D, reporting of clear details of the sample and method of recruitment/sampling; E, clear description of data collection; F, clear description of data analysis provided; G, attempts made to establish rigour of data analysis; H, discussion of ethical issues/approval details; I, inclusion of sufficient original data to support interpretations and conclusions

The included studies were conducted in the USA (n = 22), UK (n = 12), Finland (n = 2), Canada (n = 2), Norway (n = 1), France (n = 1). The majority of studies (n = 34) were conducted in inpatient settings and four were conducted within residential treatment centres in the USA. A variety of research approaches were used including experimental design (n = 4), prospective longitudinal descriptive surveys (n = 9), retrospective descriptive surveys (n = 4), cross-sectional surveys (n = 2), mixed methods (n = 4), qualitative methods (n = 8), descriptions of local initiatives and practice developments (n = 2) and clinical case reports (n = 5). Table 2 summarises the policies and guidance documents included.

**Table 2:** Policies included in phase 2

References	Title	Focus
NHS Commissioning Board [33]	NHS Standard Contract for Tier 4 Child and Adolescent Mental Health Services (CAMHS): Children’s Services	Dislocation: Fa, Fr, Ed
Department of Health [37]	National Service Framework for Children, Young People and Maternity Services: The Mental Health and Psychological Well-Being of Children and Young People (now archived)	Dislocation: St, Fa, Ed
Department of Health [24]	No Health without Mental Health: A Cross-Government Mental Health Outcomes Strategy for People of All Ages (current MH policy for England)	Dislocation: Ed, Fa; St; NL
Department of Health [38]	No Health without Mental Health: Delivering Better Mental Health Outcomes for People of All Ages	Dislocation: Ed, Fa, St
Department of Health (Kurtz) [36]	The Evidence Base to Guide Development of Tier 4 CAMHS	Dislocation: St
Royal College of Psychiatrists [58]	Acute In-Patient Psychiatric Care for Young People with Severe Mental Illness: Recommendations for Commissioners, Child and Adolescent Psychiatrists and General Psychiatrists	Dislocation: Ed, Fa
Royal College of Psychiatrists [54]	Bridging the Gaps: Health Care for Adolescents	Dislocation: Ed
Scottish Executive [24]	Child Health Support Group: Inpatient Working Group—Psychiatric Inpatient Services for Children and Young People in Scotland: A Way Forward	Dislocation: Ed, Fa, NL
YoungMinds (Street and Herts) 2005	Putting participation into practice	Dislocation: St
QNIC (Solomon et al.) [32]	Service standards (sixth edition)	Dislocation: Ed, Fa; Fr, St
Welsh Government [44]	Specialist NHS Child and Adolescent Mental Health Services: Professional Advice for Service Planners. CAMHS National Expert Reference Group	Dislocation: Ed, St
Welsh Government [41]	Together for Mental Health: A Strategy for Mental Health and Wellbeing in Wales	Dislocation: St, Ed
Welsh Government [56]	National Service Framework for Children, Young People and Maternity Services in Wales	Dislocation: Ed
Welsh Government [41]	Code of Practice to Parts 2 and 3 of the Mental Health (Wales) Measure 2010	Dislocation: Ed
NICE [25]	Antisocial Behaviour and Conduct Disorders in Children and Young People: Recognition, Intervention and Management	Dislocation: St
NICE [43]	Eating disorders: Core Interventions in the Treatment and Management of Anorexia Nervosa, Bulimia Nervosa and Related Eating Disorders	Dislocation: Ed
NICE [57]	Self-Harm: The Short-Term Physical and Psychological Management and Secondary Prevention of Self-Harm in Primary and Secondary Care	Dislocation: Ed, Fa
NICE 2012	Self-Harm: The NICE Guideline on Longer-Term Management	Dislocation: St, Fa
NICE [42]	Psychosis and Schizophrenia in Children and Young People: The NICE Guideline on Recognition and Management	Dislocation: Ed, Fa, NL
National CAMHS Support Service (no date)	Tackling Stigma: a practical toolkit	Dislocation: St

*Ad* adolescent, *Adm* admission, *CAMHS* children and adolescent mental health services, *Ch* child, *Ed* education, *Fa* family, *Fr* friends, *FU* follow-up, *HCP* health-care professional, *Id* identity, *NA* not available, *NL* normal life, *NS* not stated, *P* parent, *St* stigma

### Description of interventions or programmes

Findings from two studies investigating interventions or programmes were extracted into the category Dislocation: Education [14, 15]. The prospective cohort study [14] included data on high-school completion and educational attainment over a 20-year period, whereas the single retrospective quasi-experimental multiple time series study [15] compared a (previous) self-contained classroom format with the current rotating multiclass format for young people in a US residential training centre.

One paper by Singh et al. [16] contained findings from two studies that were extracted into the category Dislocation: Families. These rated the family-friendliness of hospital admissions prior to, and following, different types of training intended to enhance family-friendliness. In study 1 the intervention was structured role-play training and in study 2 the intervention was mindfulness training.

### Methodological quality

The methodological quality of the four experimental studies (prospective cohort study (n = 1), before-and-after studies with no control groups (n = 2), a retrospective quasi-experimental multiple time series (n = 1) was judged against the six quality criteria, and is summarised in Table 1 above.

In the two studies reported by Singh et al. [16] the sample sizes in study 1 were small, with only 18 participants before and 18 after and in study 2 the number of participants was not specified. The sample in the study by Simmerman [15] was assumed to be representative of the residential treatment centre population, although no randomisation took place. The characteristics of the young people and their families taking place in the observed mindfulness sessions for study 2 by Singh et al. [16] were not described. Little raw data was presented to verify the statistical analysis, and no ethical approval was reported for either study.

The quality of the single prospective cohort study [14] was judged to be strong, having a 20-year follow-up period. Data were first collected between 1978 and 1981 (during a period when inpatient care was different from that which exists today), and follow-up data collected 20 years later in 2001. The sample in this study was from psychiatric inpatient units in one metropolitan area of north-west USA, matched with one high school in the same area. The methodological quality of each of the eight qualitative studies was judged against nine quality criteria, and each was then further classified as being of high (n = 7), medium (n = 1) or low quality (n = 0) (see Table 1). The methodological quality of each of the 15 non-experimental studies was judged against nine quality criteria and each was then further classified as being of high (n = 6), medium (n = 7) or low quality (n = 2) (see Table 1). For the large mixed-methods studies, the individual components were quality-assessed based on study design and three were rated as high. However, the qualitative study undertaken by the Mental Welfare Commission Study [17] did not detail the study methods and so the quality could not be graded. Although the quality of research items included varied, none was excluded on quality grounds alone.

### Narrative synthesis

#### Dislocation: normal life

Five of the included studies [18–22] and three of the policy and guidance documents [23–25] addressed this area. Two subcategories were created, these being ‘Everyday life and interactions in hospital’ and ‘Missing out on life outside and transition home’.

##### Everyday life and interactions in hospital

Policy recommends that children and young people in inpatient settings are enabled to lead lives as normal as possible in the face of risks to loss of potential and unrealised hopes [23]. Access to activities was seen as important in one study [25], and in another young people spoke of the need for normalisation within inpatient units and the problems of boredom and staff shortages [22]. Young people reportedly valued everyday interactions with staff, with some preferring opportunities to engage in normal chats [21]. Others felt they were discouraged from hobbies and school-work [21], describing being confined in their rooms or denied access to everyday possessions [20] or being subjected to institutional rules including being unable to engage in normal interactions [20, 21].

##### Missing out on life outside and transition home

Home and community links were seen as important during periods of admission [24]. Young people identified feeling their normal lives as having been suspended [21], with normal rhythms, routines and relationships being lost [19]. ‘Normal’ activity outside hospital was seen as helpful to managing transitions home [21], with treatment regimes spurring young people towards discharge [18]. Post-discharge reintegration was described as seen as difficult [21].

##### Summary of dislocation: normal life

In the areas of risks to normal life, policy and guidance was sparse but did recognise that young people undergoing treatment within inpatient settings should be able to lead as normal a life as possible. Views and experiences were reported in rich detail and young people and health care professionals described boredom, stringent ward rules and routines, and a lack of opportunity for everyday interactions (CerQual—high). Feeling separated from life outside and the subsequent difficulties experienced on returning home were identified as pressing issues by some young people and health-care professionals. There were no intervention studies found that focused on the testing of actions to mitigate the risks to normal life.

#### Dislocation: identity

Three of the included studies report findings related to this area [19, 21, 26]. Two subcategories were created, these being ‘Mental health problems as identity-changing’ and ‘Responding to threats to identity’.

##### Mental health problems as identity-changing

Experience of mental health difficulties was described as identity-changing for young people with eating disorders [21, 26] and they talked of the risks of being treated in conveyor belt fashion rather than as individuals [26]. Inpatient care was described as having both unhelpful aspects (e.g., staff making assumptions about young people, and care not being individualised) and helpful aspects (e.g., being seen as unique and in need) [21].

##### Responding to threats to identity

Some young people talked about protecting their identities in the face of admission and/or receiving a diagnosis by categorising other patients, but not themselves, as ‘mentally ill’, by qualifying their diagnoses or by externalising symptoms [19].

##### Summary of dislocation: identity

In the areas of risks to identity there was no policy and guidance information. Feeling separated from life outside and the subsequent difficulties experienced on returning home were identified as pressing issues by some young people and health-care professionals (CerQual—high). Young people with eating disorders talked about mental health problems eroding their identities (CerQual—moderate), along with the experience of not being treated as individuals (CerQual—low). For other young people it was a struggle to manage threats to the sense of self during admission and treatment (CerQual—low). There were no intervention studies found that focused on the testing of actions to mitigate the risks to identity.

#### Dislocation: friends

Ten of the included studies [18–22, 26–30], one clinical case report [31] and two policies [32, 33] report findings related to this area. Two subcategories were created, these being ‘Relationships with young people outside hospital’ and ‘Relationships with young people in hospital’.

##### Relationships with young people outside hospital

Maintaining relationships with outside friends is recognised as important in policy and guidance [32, 33]. Young people in hospital were reported as valuing relationships with friends at home but could also find these difficult to sustain [19]. Some described becoming distant from their friends before admission, ascribed both to the experience of illness and to peers not understanding [21]. Admission was seen as contributing to the deterioration of friendships [19, 28, 29], with others expressing discomfort that friends visiting saw them in a mental health facility [20] or describing friendships breaking down [28]. Others talked of deliberately disconnecting from friends outside of the unit as part of a process of recovery [19]. Whilst benefits are recognised in maintaining relationships with friends at home obstacles to this are recognised [32], including rules on visiting and conflicting priorities for young people [19], and geographical distance [19, 22]. Time away from friends was also seen as helpful as a way of relieving pressures [22]. Young people recognised risks around reconnecting with friends post-discharge [19–21, 28]. In one study, ‘connectedness’ with both friends and families was found to change after being on an inpatient unit, and affected levels of depression and suicide attempts [30].

##### Relationships with young people in hospital

Living alongside other young people with similar difficulties was described as positive [18–22, 26, 27], and inpatient peer support was appreciated [19, 20, 22, 26]. On the other hand living with other young people with mental health difficulties reportedly also had negative aspects [19, 20, 22, 26, 27] including causing distress.

##### Summary of dislocation: friends

In the case of risks associated with friendships and peer relations, policy and guidance are limited to making recommendations on inpatient units having space for visitors. The evidence included in this segment of the project pointed to the difficulties (and ambivalence) young people can experience in maintaining home friendships at a distance (CerQual—high) and in reconnecting with their friends after discharge (CerQual—high). In some cases, connections with friends were significantly associated with levels of post discharge depression and suicidal ideation (CerQual—low). No intervention studies were found investigating actions to help young people in hospital maintain good relations with their peers at home. Evidence was found pointing to young people’s positive views of being with others in a similar position during hospital care and treatment, in terms of mutual support and companionship (CerQual—high). Young people also spoke of the negative aspects of living with other young people with mental health difficulties (CerQual—high). Some parents were found to be concerned about their children’s sharing of living space with other vulnerable people and at least some young people expressed ambivalence (and even fear) in their relationships with other inpatients (CerQual—low). No intervention studies were found investigating actions to promote positive peer relations among young people who were inpatients.

#### Dislocation: stigma

Six of the included studies [18–21, 34, 35] and 11 policy and guidance documents [23, 32, 36–44] address this area. Two sub-categories were created, these being ‘Young people’s experiences during admission’ and ‘Young people’s experiences post-discharge’.

##### Young people’s experiences during admission

Young people talked about specific stigmatising experiences felt to be a result of, or occurring during, inpatient admission [18–21]. They perceived stigma as flowing from the ‘outside world’ [18, 21], family members [19] and staff [21] with only a small minority reporting stigma experienced in hospital [20]. Some young people contrasted the stigmatisation felt in the outside world with the community and companionship found in hospital [18, 21].

##### Young people’s experiences after discharge

A number of factors that were found to significantly predict young people’s apprehension of stigma, including sex (being female), age at first mental health treatment (being younger at initiation of treatment) and needing greater approval from others for self-worth [35]. Six months following discharge, 70 % of young people from this study reported stigmatising experiences surrounding their mental health difficulties [34].

##### Summary of dislocation: stigma

Managing the risks of stigma and discrimination is a high priority for policy-makers. Young people felt that stigmatising experiences can occur as a result of being admitted, as well as during their inpatient stay (CerQual—moderate) and at discharge (CerQual—low). Being with similar young people can also lead to feelings of acceptance, in contrast with the experience of being rejected in the community (CerQual—low). No intervention studies were found evaluating actions to mitigate the risks of stigma or discrimination to young people admitted to mental health hospital.

#### Dislocation: education

Seventeen of the included studies [14, 15, 17–19, 21, 22, 27, 28, 45–52], one clinical case report [53], one practice initiative [31] and 14 of the policy and guidance documents [23–25, 32, 33, 37, 38, 41, 44, 54–58] addressed this area. Four subcategories were created, these being ‘Education provision and facilities’, ‘Quality of inpatient education’, ‘Academic progress’ and ‘Reintegrating with school after discharge’. Policy and guidance documents addressing these subcategories are included in each relevant section below, along with the single practice initiative.

##### Education provision and facilities

One large-scale UK study revealed that education provision for young people under 16 years of who are in mental health units is either delivered by a school integrated into the inpatient unit, or by a school located within the hospital grounds [46]. Some UK units reportedly maintain a mainstream school ethos [18], with health professionals emphasising the importance of teachers having appropriate expertise. Young people have described support during lessons in hospital [22], with a majority of teachers describing access to local schools [45]. School during admission was seen as normalising [22].

Policy is clear that inpatient units working in partnership with education services/systems is important [23, 24, 32, 37, 56–58], more specifically to maintain continuity of education provision at admission [24, 32] with a key worker/named nurse to undertake this role [24], as well as to maintain communication with the young people’s parents/carers [32]. Most inpatient units in the UK have reported good relationships with their respective education authorities [46].

Current policy also suggests those inpatients over 16 years of age should be able to continue with post-compulsory education while hospitalised [32] and that education and training providers should support students to remain on their course or hold their place open for them whenever possible [41]. In the UK, however, education provision appears to be less developed for those older than 16 years [22].

Evidence from the US includes the description of full or partial attendance at mainstream school for young people in an RTC [52]. In one US study smaller, multi-class, specialist teaching was found to be effective in increasing the amount of work young people were able to produce whilst in hospital [15].

##### Quality of inpatient education

The quality of inpatient education provided to young people in inpatient mental health hospital compared to conventional schooling has been investigated [22, 27] along with studies exploring teaching staff [18, 22, 45, 46]. Young people have been found to appreciate the supportive aspect of education [22] with only small numbers expressing concerns about the quality of schooling [27]. Additional training for teachers in child and adolescent mental health is seen to be beneficial [24, 33], with experienced teachers keeping up with training feeling that they understand the needs experienced by young people [18].

Investigations have taken place into staff/student ratios and teacher shortages [22, 45, 46]. Within England and Wales the majority of units have been reported as having a 1:3 staff-student ratio although a small number of units have reported ratios between 1:4 and 1:10 [45]. Some unit staff have said that they need more teachers [24]. Teachers, on the whole, have reported good working relationships with young people’s parents [45], though parents and young people themselves have reported instances of poor liaison [22].

##### Academic progress

Being an inpatient can have significant effects on young people’s achievements and long term goals [18, 19]. Service standards indicate that inpatient units should be registered as examination centres [32], with teachers reporting that young people have the opportunity to take their examinations [45]. Hospitalised young people have been shown to have pre-existing academic-related issues [28, 49], including below-average grades [49]. In one study 79 % on discharge reported doing the same or better in school than they had been prior to admission [51]. In investigations where young people have been followed up a number of years after hospital care to see what has happened to their educational attainment, it has been reported that they have been significantly less likely than young people without inpatient mental health experiences to complete high school, to get a bachelor’s or graduate degree [14], less likely to take up a career after discharge [47] and more likely to be expelled from school [50]. Significant predictors of academic functioning have been shown to include exposure to substances in the year post-treatment, and being a younger age at treatment [49].

##### Reintegrating with school after discharge

Re-entry and reintegration into school following discharge from hospital is reported as a major barrier to the academic progress of hospitalised young people [21, 22, 28, 45, 48], especially when an inpatient unit is far from home [22]. It is recognised in policy and guidance that education or training providers should support students to remain on their courses, or should hold places open, whenever possible [41].

Re-entry and re-integration into school has been suggested as something to consider at the point of admission [48]. There is evidence that young people enjoy the supportive aspect of education [18, 22], and a lack of education support has been associated with discharge delays [45]. Plans for re-entry into school should be made and followed through but also be flexible [28]. In studies, both health care professionals [28] and young people [21] have described school absences resulting in falling behind and young people becoming stressed during efforts to catch up. Health care professionals have suggested that students benefit from an identified, adult, support person in the school, and open communication has been identified as central to school/hospital partnerships [48]. Liaison with the young person’s mainstream school has also been suggested as vital, although some parents have described teachers not always sending homework and particular difficulties where school and hospital are geographically distant [22]. In UK inpatient units, the majority of teachers have been found to liaise with young people’s schools [45], and parents particularly see liaison with mainstream education as important for wider community reintegration [22].

Different types of school-based programme to manage transitions to school been investigated [48, 59]. Specific examples include intensive support in school and care coordination for up to 10 weeks following hospital discharge [59], and school-based re-entry and/or step-down programmes and re-entry options, with an emphasis placed on the importance of following through on interventions and asking students what is important [48].

##### Summary of dislocation: education

In policy and guidance it is clear that inpatient units should provide access to education, including appropriate education facilities/classroom space. However, no UK studies were found that looked at this area. Health care professionals, parents and young people all recognise the importance of educational provision with appropriate facilities for young people in inpatient CAMHS (CerQual—high), which is also identified as a policy and guidance priority. Smaller class sizes utilising a multiclass format with specialist teaching have been shown in a study involving young people in a RTC in the USA (GRADE—low) to be effective in increasing the amount of work young people are able to produce while in hospital. In the UK, education is provided as standard across inpatient units, but in a majority of hospitals only core National Curriculum subjects are taught (CerQual—high). Improving quality and maintaining good communication and co-ordination across hospitals and schools feature prominently in policy. Within units in the UK, varying teacher/student ratios are found in NHS and non-NHS units (CerQual—high), and good (but not universally so) relations between parents and teachers have been reported (CerQual—low).

#### Dislocation: families

Seventeen of the included studies report findings relating to the risk of dislocation from families [16–18, 21, 22, 26, 27, 29, 30, 45, 46, 50, 52, 60–63], five clinical case reports [31, 53, 64–66] and one practice initiative addressed this category [67]. Three subcategories were created, these being ‘Impact on family relationships’, ‘Family involvement’ and ‘Maintaining contact with families’. Policy and guidance documents addressing these subcategories are included in each relevant section below, along with the single practice initiative.

##### Impact on family relationships

In policy and guidance inpatient care is recognised as exerting effects on family life [36]. Improved family relationships are described as a goal of admission [33], and parent/carer support groups are recommended [32]. There is evidence that young people who are in hospital for extended periods experience homesickness [18, 22, 63], with others feeling a sense of rejection [26] or isolation [21], or that their families held negative attitudes towards them [29].

Perceptions of young people’s ‘connectedness’ with their families has been shown to change after inpatient admission, along with levels of depression and ideas about suicide [30]. Parents have expressed a need for support [18, 29], whilst in some instances family relationships have been described as breaking down [29].

##### Family involvement

Family involvement is recommended in policy and guidance [23], with working in partnership with families described as the way forward [33, 58]. This is seen as including during the development of care plans, and during the making of decisions on post-discharge care [32]. Policy and guidance refers to the value of consultation with families particularly following episodes of self-harm [43, 57]. In the case of young people with psychosis and schizophrenia, one suggestion is that alternatives to hospital admission be considered when the inpatient unit is a long way from home [25]. Training staff in inpatient units to be more friendly during the admission process by utilising role plays and mindfulness has had limited benefit [16]. Creating opportunities for families to watch films together during a young people’s hospital stay has been described as helping family engagement, and if chosen carefully, as a way of empowering families during periods of crisis [67].

A range of obstacles to family involvement have been reported by health care professionals: confidentiality (including young people’s wishes that the details of their treatment to be kept from family); parents’ own varying ability to get involved; limited time; a lack of formal structures to enable family involvement; and distance [18]. For young people whose parents are involved, benefits have been shown to include a significantly improved chance of sustaining therapeutic gains in the community [62], using after care services [61] and of avoiding readmission [50]. In one study, rehospitalisation increased when parents felt more empowered during a young person’s psychiatric treatment [60].

##### Maintaining contact with families

Inpatient units should, according to policy and guidance, have policies and procedures on visiting [32], and flexible arrangements should be in place for family contact [24]. Recommendations include family meetings within 1 week of admission, and continuing thereafter [33], along with the idea services should be offered as near to home as possible enabling frequent family visits and contact [37, 58] and appropriate family interventions [58]. When asked, young people have said they would like to keep in touch with their families [17, 21], and that whilst some units offer a flexible approach to visiting and family contact [17] this was not the same for all [21].

Some young people are placed in inpatient units located at distances from their homes, challenging regular contact with families [17, 18, 22, 27, 46]. Policy and guidance recognises that alternatives to admission should particularly be considered when hospital is a long way from where a young person lives [25]. For some young inpatients, the telephone is an important way of staying in touch [17, 18]. One finding, however, is that some young people experience the break from their usual environment as also beneficial [22]. Others describe the quality of inpatient care as more important than the distance from the hospital to their family home [17, 46]. For some parents, distance did not significantly affect the level of parent engagement or satisfaction [50].

Facilities for family visiting recommended in policy and guidance include: making available private space for family contact to take place [32, 33]; accommodating families who have to travel a significant distance [24]; and allowing parents and others to enjoy refreshments [32]. Parents [46] and young people [17, 22, 27] have both talked about the financial costs associated with admission to inaccessible locations. Some inpatient units have been described as having access to funds to financial support families receiving welfare benefits to visit [17]. Some also provide overnight provision for parents visiting from longer distances [17, 45], and provide for refreshments and privacy via use of a family room [45].

##### Summary of dislocation: family

One of the disadvantages of inpatient care recognised in policy and guidance is the effects of admission on family life. Training inpatient staff working with young people and their families through the use of role plays or mindfulness did not have a significant impact on the family-friendliness of the admission process (GRADE—low). While on an inpatient unit, young people often feel homesickness (CerQual—high) and experience a range of negative feelings (CerQual—moderate). Associations between family connectedness and post-discharge depression and suicidal ideation have been reported (CerQual—low). Some family members need additional support during their children’s admission (CerQual—low). Partnership with families during inpatient care is strongly recommended in policy and guidance. Young people whose parents do get involved make significant improvements across a range of treatment and post-discharge outcomes (CerQual—low) but health professionals report that a number of obstacles exist to enable this to take place (CerQual—low).

Whether or not families are fully involved in a young person’s care, the evidence suggests that units should have procedures on visiting and that flexible arrangements should be made for family contact. A particular risk of family dislocation is reported in instances where young people are admitted to hospitals located far from home, in terms of keeping in touch and cost (CerQual—high). For some, the quality of care at inpatient units is considered to be more important than the distance from the hospital to the family home (CerQual—moderate). Some young people also appreciated being away from the home environment (CerQual—low).

#### Dislocation: psychological development and dislocation: social

No material was included in these two categories.

### Contagion

Seven studies report findings related to the risk of contagion for young people in inpatient mental health hospital [18, 21, 22, 26, 68–70]. Two sub-categories were created: experiences of contagion, and evidence of contagion.

#### Experiences of contagion

There is evidence that health professionals and parents have concerns about young people acquiring unhelpful, destructive, behaviours during periods of admission, particularly in the areas of suicide and self-harm [18] or even just by picking up on others’ difficulties [22]. For some health care professionals, learning bad habits and witnessing disturbing and distressing events are seen as treatment failures [18].

Two studies described young people with eating disorders as being quick to copy the behaviour of those around them with the same condition [21, 26], including making comparisons with others and competing to be thin [26]. Some young people with eating disorders have described themselves as becoming more ill, in relation to their eating but also in terms of self-harm behaviours which they had not hitherto engaged in [26]. Others have described living in the same place as other people experiencing difficulties as being associated with unhelpful thoughts, comparisons and competitions [21]. However, the support of other young people during admission has also been described as positive by some (see “Dislocation: Friends”).

#### Evidence of contagion

In one study a decrease in self-harming behaviour was noted amongst young people who were inpatients who had previously engaged in this behaviour [69], and in another no evidence of contagion was found amongst young people admitted to a short-stay unit [68]. The spontaneous occurrence of self-harm amongst young inpatients not having a history of self-harm has been suggested to be low [69].

In a study which examined motivations for contagion episodes of self-harm, relieving anxiety and anger or feeling part of a group were all identified [70]. Self-cutting and bloodletting, for some in this study, was part of an initiation and group cohesion process associated with the shared experience of relief through self-harm [70].

#### Summary of contagion

The risks of young people in hospital learning harmful behaviours was a priority area for phase 2 of this project, but no policy or guidance was found addressing this. Health professionals and parents have concerns about young people acquiring unhelpful, destructive behaviours while they are inpatients (CerQual—moderate). Young people with eating disorders very quickly copy the behaviour of those around them with the same condition (CerQual—moderate). There is mixed evidence of recorded contagion in inpatient mental health facilities for young people (CerQual—low), with no fixed definition of what constitutes ‘contagion’. No evidence was located investigating actions to mitigate the risks of contagion in inpatient settings.

### Economic analysis

None of the studies included in this project reported an economic analysis or an economic evaluation of different ways of identifying, assessing and managing the less obvious risks for young people in inpatient CAMHS.

## Conclusions

In answering this project’s overarching research question a novel approach has been taken combining an initial scoping, a stakeholder consultation and an in-depth review with a narrative synthesis. A strength of the study reported here is that it has succeeded in synthesising a relatively disparate body of evidence in an area of significance to people using and working in child and adolescent mental health services. The value of the study was initially confirmed during a second stakeholder advisory group meeting, where phase 2 findings were shared and advice taken on dissemination strategies. A further strength has been the study’s sensitivity to the views and interests of stakeholders, including young people with lived experience of mental health difficulties. However, the study also has a number of limitations. First is the search for English-language only materials. A second is the inclusion of research materials from health systems around the world, without full consideration of differences in context and service configuration which have the potential to limit the transferability of findings. A further potential limitation relates to the project team’s use of umbrella terms and concepts (‘dislocation’ and ‘contagion’). Although clear definitions were developed and have been described in this article, it is acknowledged that these are broad, researcher-constructed, areas under which a diverse range of research, policy and guidance material has been subsumed.

An important contrast can be drawn between the types of risk identified in the phase 1 scoping and the types of risk identified and addressed in the in-depth phase 2 review and synthesis. Identifying and addressing clinical risks, including the risks of suicide and harm to self or others, are vitally important tasks. Inpatient child and adolescent mental health services exist partly because of their capacity to provide care to young people with high levels of need, and whose difficulties exceed the capacity of staff based in community settings [71]. However, stakeholder representatives consulted after the scoping review guided the project team towards the prioritisation of a largely different set of risks to take forward into phase 2. Inpatient CAMHS play a significant, but changing, part in complex systems of care (for a discussion, see: McDougall et al. [72], and the identification of a series of ‘less obvious’ risks under the umbrella terms ‘dislocation’ and ‘contagion’ for this project’s in-depth phase points to an awareness that inpatient admission can have wider, and long-lasting, consequences about which more needs to be known. The review summarised in this paper focused on a series of risks which are important to people with stakes in the child and adolescent mental health system, but about which little evidence exists. Service providers need to pay close attention to the identification, assessment and management of these, but a programme of research is needed to generate new knowledge underpinning the most effective and cost effective ways of achieving this.
